# Vital Statistics of Fayette County, Ky.

**Published:** 1851-11

**Authors:** 


### THE WESTERN JOURNAL.

THIRD SERIES.—VOL. VIII.—NO. V.

LOUISVILLE, NOVEMBER 1, 1851.

### VITAL STATISTICS OF FAYETTE COUNTY, KY.

We received a few days since a valuable paper, from our esteemed correspondent, Dr. W. S. Chipley, on the vital statistics of Fayette county, and regret that it came to hand too late for the present number. We shall be much pleased if the examples set by Drs. Sutton and Chipley, are followed by the physicians of Kentucky, so that the medical public may have the vital statistics of each county in the State. Essays of this kind are highly important now, but their value will increase with each revolving year, and when the census of 1860 is taken, the vital statistics of 1850 will assume their real importance. It is desirable that the medical men of Kentucky shall aid in gathering and recording such information as they can garner now, as a beginning of that great civil and social treasure—the vital statistics of the Commonwealth.

A great defect is found now in all those sources of information to which the physician must resort in an attempt to make out the vital statistics of Kentucky. A Registration law is all important, and medical men, who understand the subject should enlighten the representatives of the people, in the legislature, on the subject. When the proposition for a registration law was laid before the General Assembly, sometime since, the members of that dignified body received it as a decidedly rich joke, and they could scarcely have laughed more over the thread-bare jokes of the circus. But those who understand the subject, know how invaluable are the facts of registration. They are the meter that tells us of the condition of the people of the Commonwealth. There is no other way by which any approximation can be made to the exactitude of the information that is reflected from thorough registration, and thorough registration is perfectly practicable if the legislature shall will it. Let that body make the law, and the physicians of the State will do all the rest of the work. When the work is accomplished, the records will show infallibly the social condition of the people, and no better guarantee can be furnished for sound legislation.

The members of the General Assembly are undoubtedly willing to do all in their power to advance the true interests of the State, and if they are made to understand the character, the aim and objects of Registration, they will act upon the important subject. There is probably no member of the Legislature, who has not some acquaintance in the medical profession whose judgment and opinions on a question of this kind would have an influence for good, and we hope that every physician in the State may address himself to the work of procuring the passage of a Registration law. If the Legislature fails to pass such a law, the blame of the failure must rest upon the medical profession. If they do their duty towards the Legislature, that body will do its duty towards the people. Let the united effort of the profession, then, be seen in the enactment of the law for Registration. As a trait of its value, read the following remarks, of the Boston Medical and surgical Jcurnal. B.

"*Births, Deaths and Marriages in Massachusetts.*—In this Commonwealth, a certain class of statistics are assuming a reliable character. The eighth report to the Legislature, relating to the registry and returns of marriages, births and deaths, from May 1, 1848, to January 1, 1850, is a document of figures, which but very few men have the qualifications to construct, or the patience diligently to read and sift out the errors. Dr. Josiah Curtis, of Boston, under the direction of the Secretary of State, has systematized the town returns on this subject, and out of this mass of materials he has produced a book of 130 octavo pages, that will compare favorably with any similar report in this or other countries. Of the importance of this registration, in after times, when these United States have become old, and land-titles, the inheritance of property, and relationships, may be more essential than at the present moment, as evidence, no doubts can be entertained. It is gratifying, therefore, to perceive that effort is constantly making to improve these reports. Dr. Curtis has introduced new matter. After working through the tables, there is something to read; and it is that which Dr. C. has added, which is entitled to the reader's special thanks.

"Twenty months are embraced in the report, during which there were registered in the State, 38,313 births, 10,951 marriages, and 30,595 deaths. Within the five last years, the foreign population of Boston has increased 70.20 per cent., while the native population during the same period has decreased 2.27 per cent. Of the 63,466 foreigners in Boston, 52,923 are from Ireland; 2,666 from Germany, and 7,877 from other countries. We further learn from this publication, that there are in Boston 12,143 children of natives, and 12,132 of foreign parentage. There are 6,644 more females than males in the city.

"Within the twenty months, marriages were contracted by persons from 13 years of age to 91. Several females were married at 13. The youngest male was 16. A widow of 18 married a second husband—and cne of 59 married a fifth husband. One man of 36, and auother of 45, married a fourth time. Calvin Kilborn, of Princeton, 91, married Mrs. Susan Saunders, 70. Among females in Massachusetts, says Dr. Curtis, the chances, at the age of 20, that this interesting event will ever occur, are about 1 to 4; that is—when a female arrives at 20, and is unmarried, one-quarter of the probabilities she will be married are gone! If she passes to 25, unmarried, nearly three-quarters of her probabilities are lost. If she continue single up to 30, she has passed nine-tenths of her chances for ever becoming a wife.

"In the last five years and eight months, there were in Massachusetts 14,209 deaths by consumption. Of these, 8,453 were females, and 5,756 males.

"Dr. Curtis next treats of the laws of health, the influence of occupation upon the condition of individuals, and the laws of mortality, which subjects are very ably treated. He does himself much credit in this research, and we are glad that the Secretary has had the magnanimity, in the preface, to apprise the General Court to whom we are all indebted for this able analysis."

